# Genome-Wide Identification and Characterization of *YUCCA* Gene Family in *Mikania micrantha*

**DOI:** 10.3390/ijms232113037

**Published:** 2022-10-27

**Authors:** Weigui Luo, Nian Xiao, Feiyan Wu, Beixin Mo, Wenwen Kong, Yu Yu

**Affiliations:** 1 Guangdong Provincial Key Laboratory for Plant Epigenetics, Longhua Bioindustry and Innovation Research Institute, College of Life Sciences and Oceanography, Shenzhen University, Shenzhen 518060, China; 2 College of Physics and Optoelectronic Engineering, Shenzhen University, Shenzhen 518060, China

**Keywords:** *YUCCA*, auxin, growth and development, *Mikania micrantha*

## Abstract

Auxin is a general coordinator for growth and development throughout plant lifespan, acting in a concentration-dependent manner. Tryptophan aminotransferases (YUCCA) family catalyze the oxidative decarboxylation of indole-3-pyruvic acid (IPA) to form indole-3-acetic acid (IAA) and plays a critical role in auxin homeostasis. Here, 18 *YUCCA* family genes divided into four categories were identified from *Mikania micrantha* (*M. micrantha*), one of the world’s most invasive plants. Five highly conserved motifs were characterized in these *YUCCA* genes (*MmYUCs*). Transcriptome analysis revealed that *MmYUCs* exhibited distinct expression patterns in different organs and five *MmYUCs* showed high expression levels throughout all the five tissues, implying that they may play dominant roles in auxin biosynthesis and plant development. In addition, *MmYUC6_1* was overexpressed in *DR5::GUS Arabidopsis* line to explore its function, which resulted in remarkably increased auxin level and typical elevated auxin-related phenotypes including shortened roots and elongated hypocotyls in the transgenic plants, suggesting that *MmYUC6_1* promoted IAA biosynthesis in *Arabidopsis*. Collectively, these findings provided comprehensive insight into the phylogenetic relationships, chromosomal distributions, expression patterns and functions of the *MmYUC* genes in *M. micrantha*, which would facilitate the study of molecular mechanisms underlying the fast growth of *M. micrantha* and preventing its invasion.

## 1. Introduction

*Mikania micrantha* (*M. micrantha*) is a fast-growing and sprawling perennial vine that belongs to the *Asteraceae* family and native to tropical America [1,2]. Nowadays, it has rapidly spread in tropical Asia, including China and poses a huge threat to the native plant species by climbing and twining around other plants to block their sunlight reception and photosynthesis, eventually resulting in the death of other plants. *M. micrantha* grows extremely quickly, with a maximum mean growth rate of 20 cm each day and is named “mile-a-minute” weed [2,3]. Importantly, its plastic morphological and physiological characteristics confer strengthened survival ability even in harsh conditions [4]. As one of the most notorious invaders, *M. micrantha* has caused serious damage to natural ecosystems and substantial economic losses worldwide. Therefore, it is urgent to explore the molecular mechanisms underlying the fast-growing feature of *M. micrantha* and the biosynthetic pathways of its characteristic allelochemicals, which will lay a powerful theoretical foundation for biological control of *M. micrantha* invasion.

Auxin is one of the major phytohormones and orchestrates the developmental processes throughout the life cycle of plants. Intriguingly, auxin modulates plant growth and development in a dosage-dependent manner, which largely depends on the radial auxin concentration gradients across plant tissues [5,6,7]. Therefore, it is vital to finetune the auxin level by strictly controlling auxin metabolic pathways in plant cells for proper development and response to environmental stimuli [8,9]. There are three naturally occurring compounds with direct auxin activity in plants: indole-3-acetic acid (IAA), 4-chloroindole-3-acetic acid (4-Cl-IAA) and phenylacetic acid (PAA), among which IAA is best characterized [10,11]. IAA can be produced in both tryptophan (Trp)-dependent and Trp-independent manners [5,12,13]. In the Trp-dependent pathway, Trp is first converted to indole-3-pyruvic acid (IPA) by the TRYPTOPHAN AMINOTRANSFERASE OF ARABIDOPSIS 1 (TAA1) and TAA1-RELATED proteins (TARs). Then IPA is oxidative decarboxylated to produce IAA by YUCCA (YUC) family of flavin monooxygenases (FMO) and this is the irreversible rate-limiting reaction for IAA biosynthesis [12,14,15]. In *Arabidopsis*, 3 *AthTAA1/TARs* and 11 *AthYUC* genes have been identified. Unlike the broad expression of *AthTAA1/TARs* genes [16], *AthYUC* genes exhibit distinct organ-specific expression patterns [5,9,17]. Genetic and molecular evidence has shown that the spatiotemporal expression of specific *AthYUC* genes is required for normal plant growth and proper organ development [5,9]. Due to the strong functional redundancy of *AthYUC* genes, the single mutants of *AthYUC* genes show none or only subtle developmental defective phenotypes, but double or multiple *yuc* mutants bypassing functional redundancy display marked reductions in IAA levels and severe developmental defects in *Arabidopsis*, such as abnormal leaf and floral patterning, altered vasculature formation, aberrant embryo patterning, abnormal apical hook formation, reduced stature, defective root growth and gravitropic response [7,18,19,20,21]. Among the 11 *AthYUC* genes, four of them (*AthYUC1*, *AthYUC2*, *AthYUC4* and *AthYUC6*) have been determined to play essential roles in auxin biosynthesis and plant development. The triple and quadruple mutants of these four *AthYUC* genes all display severe defects in floral patterning, vascular formation and other developmental phenotypes [18,19]. Besides, *yuc2yuc6* double mutant fails to produce viable pollens grains, indicating that *AthYUC2* and *AthYUC6* are two key genes essential for male fertility [22]. Interestingly, overexpression of *YUC* genes and excessive auxin content also lead to abnormal phenotype. It is found that dominant activation mutant *yuc6-1D* and *35S::AthYUC6* transgenic plants possess elevated auxin levels in leaves and perform dramatic a longevity phenotype [18,23,24]. *35S::AthYUC4* transgenic plants exhibit epigastric cotyledons and elongated hypocotyls in seedlings accompanied with twisted cauline leaves, narrow rosette leaves, long petioles and increased apical dominance in adult plants [18,25]. Overexpression of *AthYUC8* and *AthYUC9* results in aberrant secondary growth of the stem and narrow leaves in *Arabidopsis* [26].

*YUC* homologs are identified across the genomes of vascular and nonvascular plants [17,27] and the conserved functions of *YUC* homologs have been reported in maize [28,29], rice [30,31,32] and *Marchantia polymorpha* [33], etc. However, there is no report on the basic knowledge of *YUC* family genes in *M. micrantha*, which may be the key regulators for its fast growth. In the present study, we performed a genome-wide survey of the *MmYUC* gene family in *M. micrantha* and identified 18 putative *MmYUCs*. Multiple sequence alignments, phylogenetic relationships, conserved motifs, chromosome distributions and gene duplication analysis of these *MmYUC* genes were performed. The expression patterns of *MmYUC* genes in five different tissues were also determined based on RNA-seq data. In addition, overexpression of *MmYUC6_1* in *Arabidopsis* enhanced IAA biosynthesis and the transgenic plants displayed an abnormal phenotype related to over-accumulated auxin level, indicating that *MmYUC6_1*, probably also other *MmYUCs* genes, had an essential role in maintaining auxin homeostasis in *M. micrantha*. Collectively, our study provided a necessary foundation for analysis of auxin biosynthesis and would benefit for exploring the underlying mechanisms of the rapid growth of *M. micrantha*, as well as the biological control of this invasive species.

## 2. Results

### 2.1. MmYUC Gene Family Analysis in Mikania micrantha

**Figure 1:**
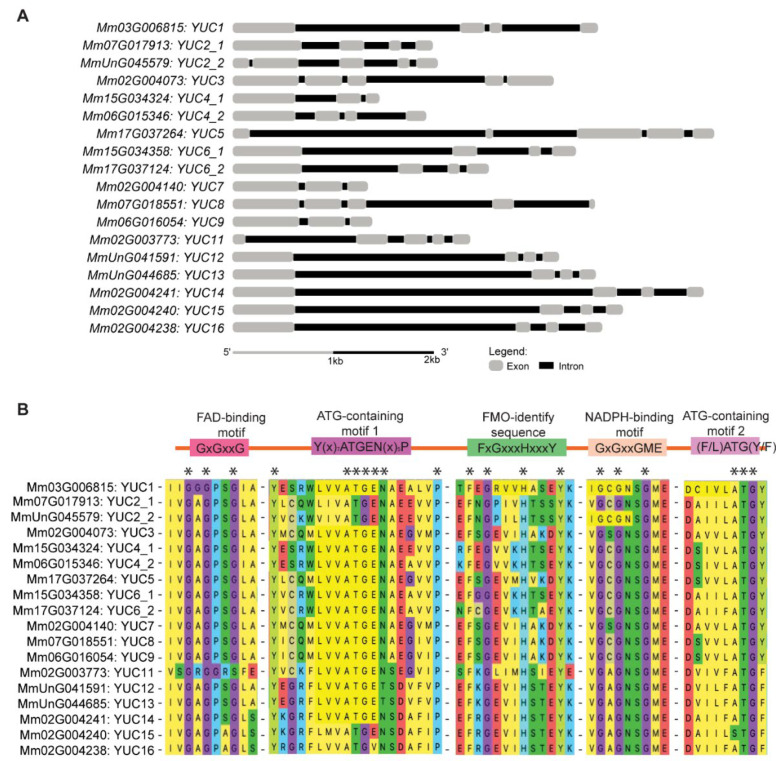
*MmYUC* members identified in *M. micrantha* genome. (**A**) Structure of *MmYUC* genes. The exons and introns are represented by grey boxes and black lines, respectively. (**B**) Alignment of conserved domains in MmYUCs. Stars (*) indicate the conserved amino acids in the motif.

To identify putative *MmYUC* genes in *M. micrantha*, we searched the *M. micrantha* genome [34] using the amino acid sequences of *Arabidopsis* AthYUC proteins as queries. In total, 18 candidate genes were filtered out. According to the protein sequence similarities among AthYUCs and MmYUCs, *MmYUC* genes were divided into four clades in the phylogenetic tree (Figure S1) and named as *MmYUC1*, *MmYUC2_1*, *MmYUC2_2*, *MmYUC3*, *MmYUC4_1*, *MmYUC4_2*, *MmYUC5*, *MmYUC6_1*, *MmYUC6_2*, *MmYUC7*, *MmYUC8*, *MmYUC9*, *MmYUC11*, *MmYUC12*, *MmYUC13*, *MmYUC14*, *MmYUC15* and *MmYUC16*, respectively. An exon-intron composition map was drawn to characterize the structural diversities of *MmYUC* genes (Figure 1A), which revealed that members in the same clade shared similar intron-exon compositions, numbers and lengths and they may have a close evolutionary relationship. More detailed information of these putative *MmYUC* genes is listed in Table S1.

The distribution of *MmYUC* genes on the 19 chromosomes was analyzed based on the genome annotation (Figure S2). 15 *MmYUC* genes were successfully mapped to eight chromosomes, while the remaining three *MmYUC* genes (*MmYUC2_2*, *MmYUC12* and *MmYUC13*) were not mapped to any position across the assembled chromosomes, probably due to the inaccurate genome assembly. As for the mapped *MmYUCs*, they randomly distributed on the chromosomes, but we did notice that some *MmYUC* genes located close to each other. For example, among the 6 *MmYUC* genes on Chr02, *MmYUC14*, *MmYUC15* and *MmYUC16* extremely closely arranged. Together with the same clade and similar gene structures, it is possible that these three *MmYUC* genes exhibit similar expression pattern and redundant biologic functions, both of which remain to be explored.

Previous studies have showed that YUCCA family members share highly conserved motifs, including FAD-binding motif (GxGxxG), ATG-containing motif 1 [Y(x)_7_ATGEN(x)_5_P], FMO identifying motif (FxGxxxHxxxY/F), NADPH-binding motif (GxGxxG) and ATG-containing motif 2 [(F/L)ATG(Y/F)]. To examine whether the proteins encoded by these putative *MmYUC* genes contain these conserved motifs, we analyzed the deduced amino acid sequences using the domain analysis program Pfam (Protein family, http://pfam-legacy.xfam.org, accessed on 5 August 2021) [35]. The results showed that these 18 putative MmYUC proteins all contain the conserved FMO identifying motif, FAD-binding motif, NADPH-binding motif (Figure 1B), suggesting that the MmYUC proteins would exert conserved functions. Nevertheless, some differences were observed in the ATG-containing motif 1 of MmYUC16 and ATG-containing motif 2 in MmYUC15, which could contribute to the functional divergences.

### 2.2. Phylogenetic Analysis of MmYUC Gene Family

**Figure 2:**
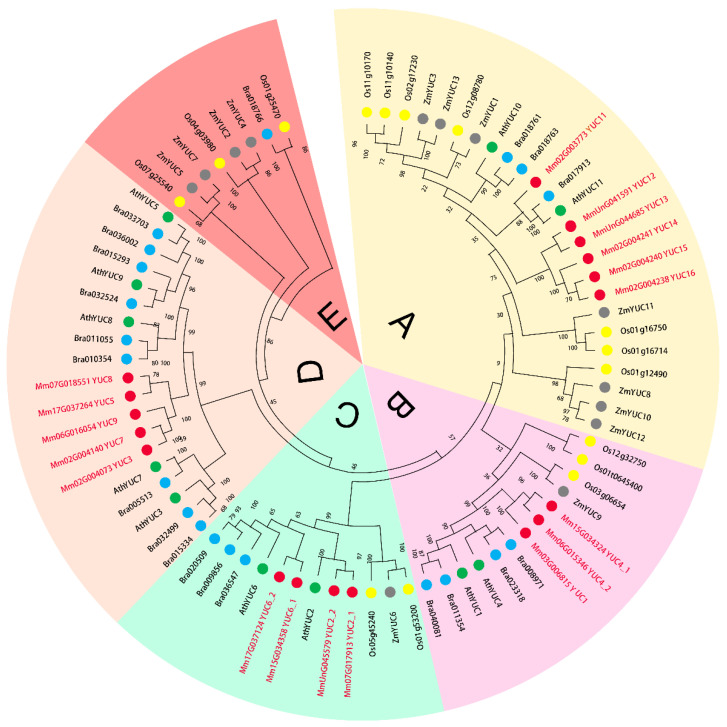
Phylogenetic relationships of *YUC* genes among *Arabisopsis thaliana* (green), *Oryza sativa* (yellow), *Brassica rapa* (blue), *Zea mays* (grey) and *M. micrantha* (red).

In order to explore the phylogenetic relationship of *YUC* genes between *M. micrantha* and other plant species, we constructed a combined phylogenetic tree using the amino acid sequences of YUC proteins from *M. micrantha*, *Oryza sativa*, *Arabidopsis thaliana*, *Brassica rapa* and *Zea mays*. As shown in Figure 2, YUC proteins from these five species were grouped into 5 clades (A, B, C, D and E). Consistent with our previous analysis, 18 MmYUC proteins fell into clades A-D, respectively, while no MmYUC was found in the clade E. The proteins in same clade may undergo close evolutionary process and possess similar function. It was reported that *Os03g06654* (*OsYUC1*), a ubiquitously expressed gene encoding a key FMO enzyme in rice, was involved in IAA synthesis and plant growth and development [31]. MmYUC1, MmYUC4_1 and MmYUC4_2, were found in the same clade with OsYUC1 (Figure 2), indicating that these MmYUCs may play a role in IAA biosynthesis and the rapid growth of the *M. micrantha*. MmYUC6_1 and MmYUC6_2 belonged to clade C and were highly close to AthYUC6 (Figure 2), a gene specially and highly expressed in *Arabidopsis* flowers [18], suggesting that MmYUC6_1 and MmYUC6_2 may function in the flower development of *M. micrantha*. In addition, we found that both MmYUC2 and MmYUC6 had two members, which were closely related to AthYUC2 and AthYUC6, respectively (Figure 2), implying that they might undergo replication during the evolution.

### 2.3. Expression Pattern of MmYUC Genes in Different Tissues

**Figure 3:**
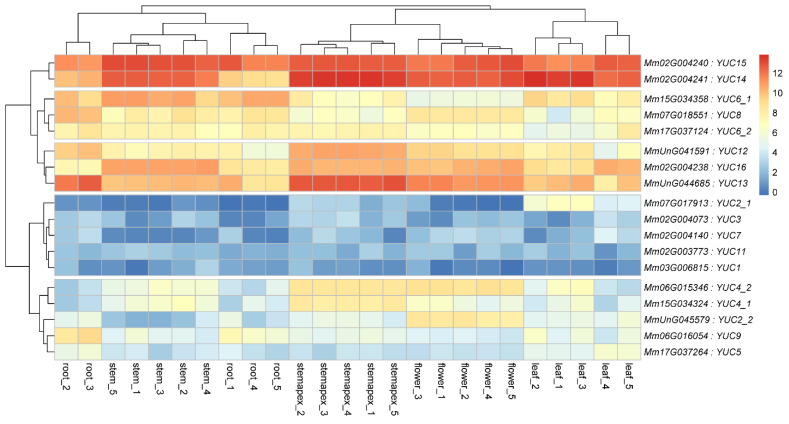
Heat map of the expression patterns of *MmYUC* genes in different tissues.

To explore the specific functions of *MmYUC* genes, we analyzed the expression profiles of *MmYUCs* in five different tissues of *M. micrantha*, including root, leaf, stem, flower and stemapex, using the published RNA-seq data (BioProject ID PRJNA528368) [34] (Figure 3). Based on their general expression levels among the five tissues, the 18 *MmYUC* genes could be divided into five groups. Except for *MmYUC1_1*, *MmYUC2_1*, *MmYUC3*, *MmYUC7* and *MmYUC11*, other genes were expressed in almost all tissues, but the expression patterns of each gene were different. *MmYUC14* and *MmYUC15* showed the highest expression levels in almost all analyzed tissues (Figure 3), suggesting that they might have universal roles during plant growth and development. *MmYUC12*, *MmYUC13*, *MmYUC16*, *MmYUC6_1*, *MmYUC6_2* and *MmYUC8* were also detected in all analyzed tissues with lower expression levels (Figure 3). It was notable that *MmYUC13* was expressed at a higher level in stemapex than other tissues (Figure 3), implying that *MmYUC13* could play a key role in stem elongation. In summary, *MmYUC* members showed distinct expression patterns in different tissues, suggesting that they could coordinately regulate the synthesis of auxin in *M. micrantha*. To validate whether the transcriptome analysis results could reflect the real expression profiles of these *MmYUC* genes, the expression levels of *MmYUC6_1* and *MmYUC6_2* in leaf, stem and flower tissues were further examined by RT-qPCR. As expected, *MmYUC6_2* showed a higher expression level in stem and flower than other tissues (Figure S3). The consistent expression profiles between the transcriptome and RT-qPCR indicated that the transcriptome analysis was reliable.

**Figure 4:**
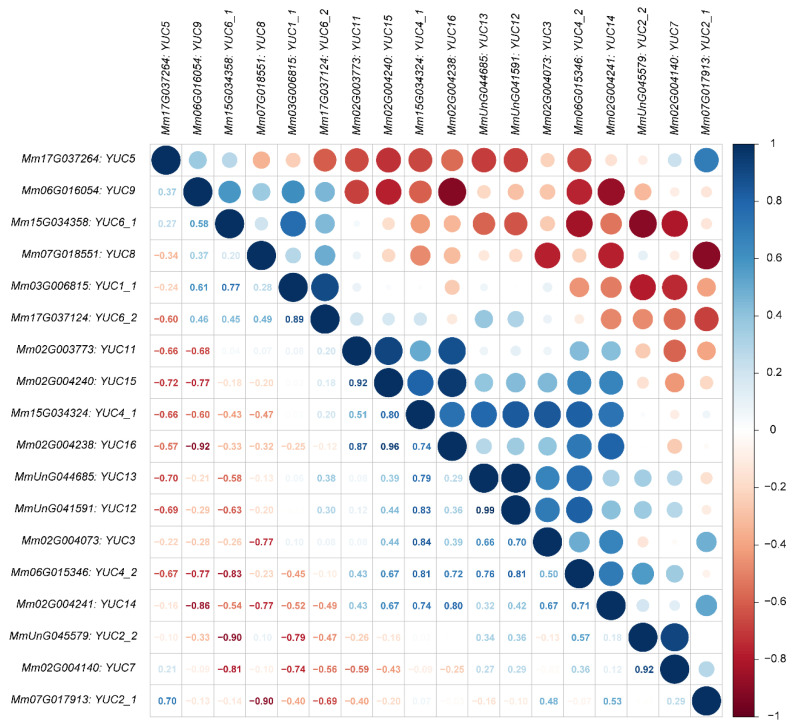
The correlation of gene expression patterns between every two *MmYUC* genes. Red color and blue color represent negative and positive correlation, respectively.

In addition, we analyzed the expression correlation between every two *MmYUC* genes in five tissues. As shown in Figure 4, almost half of the genes in different tissues were positively correlated, among which *MmYUC12* and *MmYUC13*, as well as *MmYUC15* and *MmYUC16*, were significantly positively correlated. Together with their close relationship in the phylogenic tree (Figure 2), this indicated that *MmYUC12* and *MmYUC13*, as well as *MmYUC15* and *MmYUC16*, may have a redundant function. In contrast, *MmYUC9* and *MmYUC16*, *MmYUC2_2* and *MmYUC6_1*, as well as *MmYUC2_1* and *MmYUC8*, were significantly negatively correlated (Figure 4), suggesting that the two genes may play opposite roles in *M. micrantha*.

### 2.4. Heterologous Expression of MmYUC6_1 in Arabidopsis

**Figure 5:**
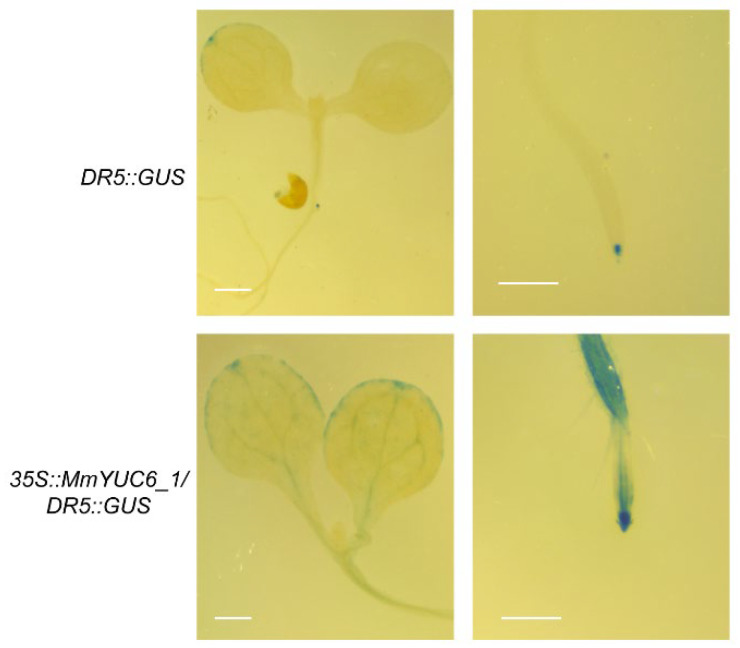
GUS staining of *DR5::GUS* and *35S::MmYUC6_1/DR5::GUS* transgenic plants. Bar scale, 1 mm.

The transgenic plant *DR5::GUS* is a commonly used auxin-reporter line for approximate assessment of auxin concentration and distribution in plants [36,37,38,39]. To further characterize the potential functions of *MmYUCs* in auxin biosynthesis and accumulation, we overexpressed *MmYUC6_1* in the *DR5::GUS* line in *Arabidopsis* and observed the developmental phenotype of homozygous *35S::MmYUC6_1/DR5::GUS* plants in T3 generation. The reason we chose *MmYUC6_1* was that this gene showed moderate expression levels among the *MmYUC* gene family and it also exhibited distinct expression pattern in the five tissues. In addition, its homolog *AthYUC6* has been reported to be essential for both vegetative and reproductive development in *Arabidopsis* [18,23,24]. The GUS signal was first analyzed in *DR5::GUS* and *35S::MmYUC6_1/DR5::GUS* transgenic lines. As expected, overexpression of *MmYUC6_1* led to much stronger GUS signal in both cotyledons and roots of *35S::MmYUC6_1/DR5::GUS* seedlings than that in *DR5::GUS* (Figure 5), suggesting that *MmYUC6_1* significantly elevated IAA levels in *Arabidopsis*.

**Figure 6:**
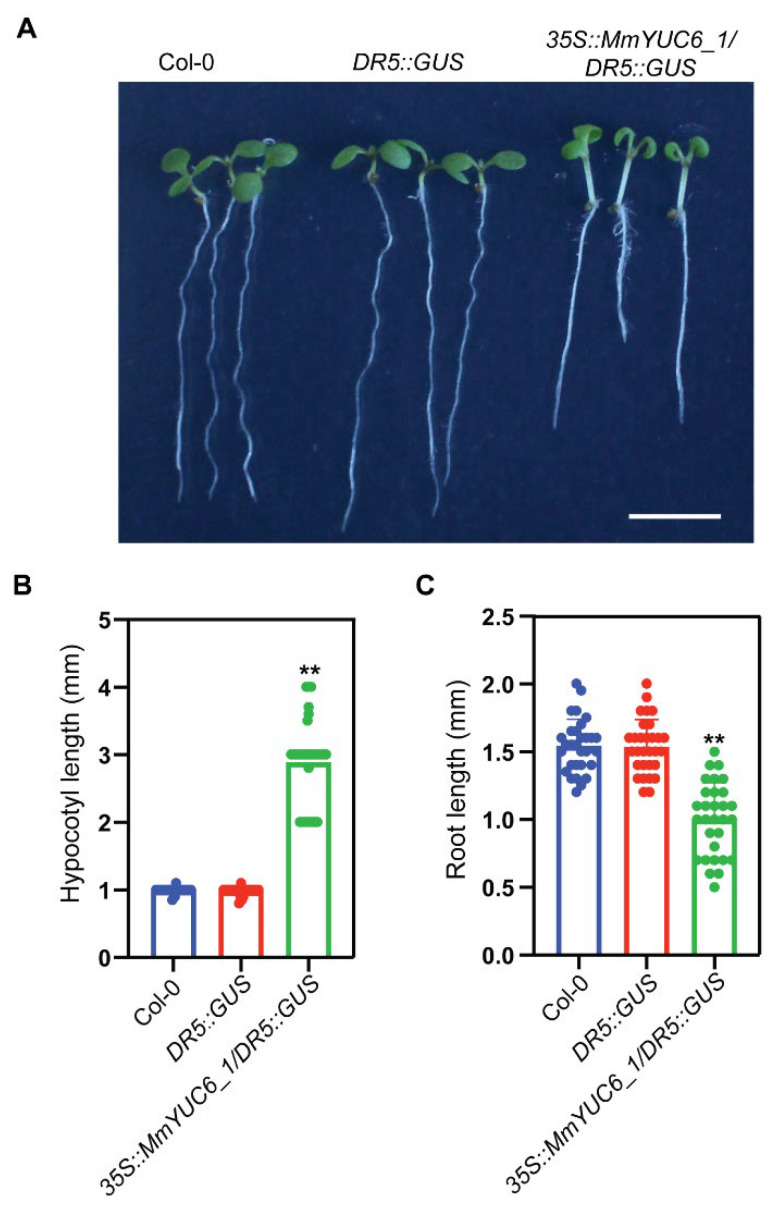
Phenotype of *DR5::GUS* and *35S::MmYUC6_1/DR5::GUS* plants. (**A**) Phenotypes of 7-days-old seedlings. Bar scale, 5 mm. (**B**) Quantification analysis of the hypocotyl lengths of *DR5::GUS* and *35S::MmYUC6_1/DR5::GUS* seedlings (n = 30). (**C**) Quantification analysis of the root lengths of *DR5::GUS* and *35S::MmYUC6_1/DR5::GUS* seedlings (n = 30). ** represents the significant difference (*p* value < 0.01).

As a double-edged sword, a certain amount of auxin is required for proper cell elongation; however, over-accumulated auxin will inhibit cell elongation and the growth of plant organs, especially the root. Consistently, the *MmYUC6_1* overexpression plants exhibited elongated hypocotyls and shortened roots, which were typical auxin over-accumulated phenotypes (Figure 6A). Quantification analysis revealed that the length of hypocotyls from *35S::MmYUC6_1/DR5::GUS* transgenic plants was increased by about 3-fold and the root length of *35S::MmYUC6_1/DR5::GUS* was reduced by around 30%, compared to that of Col-0 and *DR5::GUS* plants (Figure 6B,C). Taken together, these results proved that *MmYUC6_1* played a critical role in IAA biosynthesis and may also be essential for the fast-growing characteristic of *M. micrantha*.

## 3. Discussion

As one of the most notorious invaders, *M. micrantha* poses a great threat to native species and natural ecosystems. However, the genes connected with its physiological characteristics and fast growth remain largely unknown. *YUC* gene family plays a vital role in IAA biosynthesis to maintain auxin concentration gradients and regulate plant growth and development. In this study, we predicted and revealed the comprehensive features of the *MmYUC* gene family in *M. micrantha*, which may be important to the fast growth and strong reproducibility of this species, through bioinformatics analysis for the first time. Among the 18 *MmYUC* putative genes, the homolog of *Arabidopsis AthYUC10* was not found and *MmYUC12/13/14/15/16* had no homologs in *Arabidopsis* or other species analyzed in this study, implying that these *MmYUCs* may be *M. micrantha*-specific. It is well-known that *AthYUC2*, *AthYUC4* and *AthYUC6* are essential for both vegetative and reproductive development in *Arabidopsis* [18,19]. Notably, two homologous *MmYUC* genes were identified for *AthYUC2*, *AthYUC4* and *AthYUC6*, respectively, suggesting that these *MmYUCs* may also play a critical role in *M. micrantha* growth and development processes and the duplication of these genes during the evolution could be a canny strategy to reinforce the invasive ability of *M. micrantha*. In addition, based on the phylogenetic analysis, *MmYUC* genes deservedly clustered in one branch within each clade, which greatly differed from other species and could be one of the specificities of this species.

Auxin biosynthesis pathways vary greatly among different organs and tissues to satisfy the demand of disparate auxin-response levels. *YUC* gene family members are expressed in distinct patterns to perform tissue-specific functions. There are two *MmYUC* members, *MmYUC14* and *MmYUC15*, displaying widely high expression among all five detected tissues, indicating their dominate and universal role in auxin biosynthesis in *M. micrantha*. Besides, it was reported that four of the *Arabidopsis AthYUC* genes (*AthYUC1*, *AthYUC2*, *AthYUC4* and *AthYUC6*) were expressed mainly in the inflorescence apex and flowers to regulate reproductive organs development [18,19]. However, homology of these genes in *M. micrantha* (*MmYUC1*, *MmYUC2_1*, *MmYUC2_2*, *MmYUC4_1*, *MmYUC4_2*, *MmYUC6_1* and *MmYUC6_2*) exhibited only moderate, or even low, expression levels in flowers, which was different from *Arabidopsis*. The difference may be resulted from the duplication and functional redundancy of *MmYUC* genes. Furthermore, it is worth noting that, as a vine specie, *M. micrantha* has a special organ-stemapex, which is able to climb and wrap other plants. Cell expansion and division in stemapex are much faster than in other tissues, which could rely on the performance of auxin. Based on the expression patterns of *MmYUCs* (Figure 3), in addition to *MmYUC14* and *MmYUC15*, the two widely and highly expressed genes, *MmYUC13*, *MmYUC12* and *MmYUC16,* also exhibited high expression levels in stemapex, implying that they may contribute to auxin biosynthesis in stemapex and be involved in the fast-growing physiological features of stemapex. The expression pattern analysis of *MmYUC* genes undoubtedly provide important information for future research on *MmYUC* gene functions and give assistance in predicting spatiotemporal distribution of auxin in *M. micrantha*.

Auxin acts as a double-edged sword in plant growth and development, therefore the precise content of auxin is necessary to ensure normal morphological establishment of plants. Both mutation and overexpression of *YUC* genes would disrupt the auxin homeostasis, invoking a series of growth defects [18,19,24,25]. Referring to the previous studies, it is not difficult to find that not only overexpression of *AthYUC* genes leads to abnormal phenotype, but heterologous expression of other *YUCs*, such as strawberry *FaYUC1* [40], wheat *TaYUC10* [41], cucumber *CsYUC11* [42] and apple *MdYUC8* [43] also resulted in various defects in vegetative and reproductive processes in *Arabidopsis*. Similar examples can also be validated in other species, for example, overexpression of *FvYUC6* caused delayed flowering and male sterility in woodland strawberry [44]. In this study, *MmYUC6_2* was overexpressed in *Arabidopsis* and, as expected, the transgenic plants showed typical auxin-overproducing phenotypes, such as elongated hypocotyl and shortened root in seedlings. Collectively, we speculated that knock-out or overexpression of *MmYUC* genes in *M. micrantha* may affect its growth and reproduction ability and could be applied to prevent the widespread of *M. micrantha*.

## 4. Conclusions

In this study, 18 putative *MmYUC* genes were identified in *M. micrantha* and their features were systematically analyzed, including gene structures, phylogenetic characteristics, expression patterns and functional characterization by heterologous expression in *Arabidopsis*. Phylogenetic analysis and spatial-temporal expression pattern analysis revealed that the *MmYUC* genes were conserved in function and evolution with other species, suggesting that they may be involved in IAA biosynthesis, vegetative and reproductive development of *M. micrantha*. In addition, one *MmYUC* gene, *MmYUC6_1*, was overexpressed in *Arabidopsis* to investigate its function. Heterologous expression of *MmYUC6_1* could promote auxin accumulation and lead to a typical hyper-auxin performance in *Arabidopsis*. Taken together, these findings provide important information for the elucidation of the function of *MmYUC* genes in *M. micrantha* growth and development processes, relevant for biological prevention and control of this aggressive invader.

## 5. Materials and Methods

### 5.1. Identification and Sequence Homology Analysis of Candidate MmYUC Gene Family Members in M. micrantha

To identify putative *MmYUC* genes in *M. micrantha*, the protein sequence of 11 *Arabidopsis thaliana AthYUC* genes were obtained from TAIR (https://www.arabidopsis.org, accessed on 17 June 2020) and used as the query sequences to screen all candidate proteins in *M. micrantha* with the Basic Local Alignment Search Tool (http://blast.ncbi.nlm.nih.gov, accessed on 17 June 2020). Parameters was set as follows: E-value (1 × 10^−10^), num_threads (30), outfmt (0), num_alignments (5). The filtered target genes were subjected to the Hidden Markov Model (HMM) database with E-value threshold of 0.01 from the Pfam protein family database (http://pfam-legacy.xfam.org, accessed on 5 August 2021). Highly conserved motifs (or amino acid residues) of the candidate *MmYUC* genes were identified with multiple sequence alignments by the ClustalW program [45] at the SIB Bioinformatics Resource Portal (https://myhits.sib.swiss/cgi-bin/clustalw, accessed on 5 August 2021). Sequence alignments were visualized using the BOXSHADE 3.21 program (http://www.ch.embnet.org/software/BOX_form.html, accessed on 7 August 2021).

### 5.2. Phylogenetic Analysis

The amino acid sequences of YUC proteins from *Arabisopsis thaliana*, *Oryza sativa*, *Brassica rapa* and *Zea mays* were obtained from TAIR (https://www.arabidopsis.org/, accessed on 17 June 2020), RGAP (http://rice.plantbiology.msu.edu/, accessed on 17 June 2020), Brassica Database (http://brassicadb.org, accessed on 17 June 2020) and MaizeGDB (https://maizegdb.org/, accessed on 17 June 2020), respectively. MEGAX software (v.11) was used for phylogenetic analysis using the neighbor-joining (NJ) method under the Jones-Thornton-Taylor (JTT) model with 1000 replicates of bootstrap based on the alignment results from ClustalW program with the following parameters: substitution type, poisson model, uniform rates, partial deletion. The phylogenetic tree only showed branches with a bootstrap consensus > 50. Based on the multiple sequence alignment and phylogenetic analysis, the *MmYUC* genes were assigned to different groups and subgroups according to the classification of *AthYUC* genes reported previously.

### 5.3. Chromosomal Location, Gene Duplication and Selection Pressure Analysis

The chromosomal location information of all *MmYUC* genes was obtained from the project described by Liu et al. (2020). The physical map of *MmYUC* gene family was generated using TBTools software [46]. To detect segmental and tandem duplication events, MCScanX with the parameters (match score (>50); match size (5); gap penalty (−1); overlap window (5); E-value: 1 × 10^−5^; max gaps (25)) were used and the result was visualized with TBTools software.

### 5.4. Analysis of MmYUC Genes Expression Patterns in the RNA-seq Data

The RNA-seq data of *M. micrantha* root, stem, flower, leaf and stemapex were collected from the published paper (BioProject ID PRJNA528368) [34]. The expression profiles of the *MmYUC* genes were estimated by RPKM values (reads per kilobase per million mapped reads) and a heat map was constructed with R packages (Pheatmap and Stats) to show the differentially expression profiles.

### 5.5. RNA Isolation and qRT-PCR Analysis

Tissue samples were collected and frozen immediately in liquid nitrogen for RNA extraction. Total RNA was isolated with AG RNAex Pro RNA Regent (Accurate Biology, Cat#AG21101) and 5 μg RNA was reverse-transcribed into cDNA with SuperScript IV First-Strand Synthesis System (Invitrogen, Cat#18091050, Waltham, MA, USA) according to the manufacturer’s instructions. qRT-PCR was performed in a BIORAD qPCR instrument using ChamQ SYBR qPCR Master Mix (Vazyme, Cat#Q311, Nanjing, China). Data was normalized to *ACTIN II* expression by the cycle threshold (CT) 2^−ΔΔCT^ method as described before and analyzed by Prism7 software (Graphpad, San Diego, CA, USA). Primers used are listed in Supplementary Table S2.

### 5.6. Heterologous Expression of MmYUC6_1 in Arabidopsis

The coding sequences of *MmYUC6_1* was cloned from *M. micrantha* cDNA and inserted into the vector via infusion-clone to construct the *35S::MmYUC6_1* vector. The plasmids were transferred into *Agrobacterium tumefaciens* strain GV3101 and transformed into *DR5::GUS* plants by floral dip method. T1 seeds were screened in the 1/2 MS (Murashige Skoog) plate containing 0.1% hygromycin. 7-day-old seedlings were transplanted into soil and cultivated in the greenhouse setting with cycles of 16 h light/8h dark at 22 °C. Primers used are listed in Supplemental Table S2.

### 5.7. GUS Staining

7-day-old seedlings of *DR5::GUS* and *35S::MmYUC6_1/DR5::GUS* transgenic plants were collected and submerged in GUS staining solution (0.1% Triton X-100, 10 mM EDTA, 2 mM ferrocyanide, 2 mM ferricyanide, 100 mM sodium dibasic phosphate, 100 mM sodium monobasic phosphate and 4 mM 5-bromo-4-chloro-3-indolyl-β-glucuronide) with the assist of vacuum for 15 min. After incubating at 37 °C for 48 h or until blue color was visible, chlorophyll was completely removed by 95% ethanol. Then the stained samples were photographed using a stereomicroscope (Leica S8 APO, Wetzlar, Germany) with a digital camera (Nikon D7000, Tokyo, Japan).

## Supplementary Materials

The following supporting information can be downloaded at: https://www.mdpi.com/article/10.3390/ijms232113037/s1.
